# Primary Spinal Marginal Zone Lymphoma Presenting With Spinal Cord Compression: A Case Report

**DOI:** 10.7759/cureus.110109

**Published:** 2026-06-02

**Authors:** Mohammed Amine Guerrouaz, Soufiane Berhili, Mohamed Moukhlissi, Karim Bahhous, Ibrahim Ait Ouaggou, Loubna Mezouar

**Affiliations:** 1 Department of Radiation Oncology, Faculty of Medicine and Pharmacy of Oujda, Mohammed First University, Mohammed VI University Hospital, Oujda, MAR; 2 Department of Radiation Oncology, Mohammed VI University Hospital, Oujda, MAR

**Keywords:** malt lymphoma, marginal zone lymphoma, radiotherapy, spinal compression, spinal lymphoma

## Abstract

Extranodal marginal zone lymphoma (EMZL) is an indolent B-cell lymphoma that typically arises in mucosal or extranodal tissues. Spinal involvement is exceptionally rare and may present with spinal cord compression, posing diagnostic and therapeutic challenges. We report the case of a 70-year-old man who presented with progressive paraparesis following a four-month history of lumbosciatic pain. MRI revealed an epidural and paravertebral mass extending from L2 to L4 with vertebral body destruction and spinal canal invasion. Urgent decompressive laminectomy with subtotal resection was performed. Histopathology and immunohistochemistry confirmed EMZL. Staging excluded systemic disease. The patient received adjuvant radiotherapy (24 Gy in 12 fractions) using volumetric modulated arc therapy (VMAT). Treatment was well tolerated, with neurological recovery and a good clinical outcome. Spinal EMZL is a rare cause of spinal compression. Accurate diagnosis requires integrated histopathologic and immunophenotypic evaluation. Combined surgical decompression and standard-dose radiotherapy can provide effective local control and favorable outcomes.

## Introduction

Extranodal marginal zone lymphoma (EMZL) is an indolent mature B-cell lymphoma accounting for approximately 5%-10% of all non-Hodgkin lymphomas [1,2]. It predominantly affects older adults, with a median age at diagnosis around the sixth decade of life, and is frequently associated with chronic antigenic stimulation related to autoimmune or infectious conditions [1,3]. EMZL most commonly arises in extranodal mucosal sites, particularly the stomach, which represents the most frequent location, followed by the salivary glands, thyroid, lung, and ocular adnexa [3,4]. The disease usually follows an indolent clinical course with prolonged survival, although local relapse or late recurrence may occur. Primary spinal or epidural involvement remains exceptionally rare [4]. Because spinal EMZL may mimic other epidural or vertebral neoplasms clinically and radiologically, accurate diagnosis requires careful integration of imaging, histopathology, and immunophenotypic findings. Reporting additional well-characterized cases may improve diagnostic recognition and therapeutic decision-making in this uncommon presentation.

Given the exceptional rarity of primary spinal EMZL and the diagnostic challenges posed by spinal epidural lymphoid lesions, documenting additional well-characterized cases may help improve diagnostic recognition and therapeutic decision-making.

## Case presentation

A 70-year-old man presented in October 2025 with progressively worsening low back pain associated with lower-limb weakness and gait impairment evolving over several weeks. The patient had no significant past medical history, autoimmune disease, or prior malignancy. He initially received symptomatic treatment. One month later, he developed rapidly progressive paraparesis leading to loss of ambulation, prompting urgent neurosurgical evaluation.

Lumbar MRI revealed a poorly circumscribed epidural and paravertebral soft tissue mass extending from L2 to L4, hypointense on T1, hyperintense on T2/STIR, and avidly enhancing after gadolinium administration. The lesion measured 60 × 46 mm axially and 67 mm craniocaudally (Figure 1A). It invaded the right L2 neural foramen with extension into the spinal canal, displacing nerve roots to the left (Figure 1B). At the L3 level, the lesion occupied nearly the entire spinal canal with contralateral (left) foraminal extension (Figure 1C). Lytic destruction of the L3 vertebral body with superior endplate collapse was noted (Figure 1D). The mass infiltrated the right paravertebral soft tissues and right psoas muscle between L2 and L4 (Figure 1B). Lumbar lordosis was preserved.

**Figure 1:**
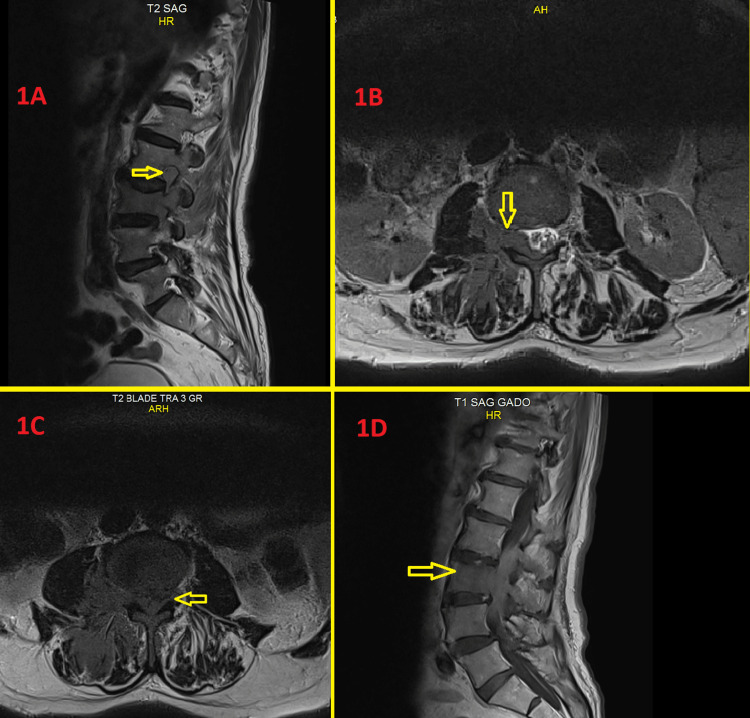
Lumbar spine MRI demonstrating an epidural and paravertebral lymphoma causing spinal canal compression. (A) Sagittal T2-weighted MRI showing an extensive epidural and paravertebral soft-tissue mass extending from L2 to L4, hyperintense on T2, causing marked spinal canal compromise. (B) Axial T2-weighted image at the L2 level demonstrating tumor invasion of the right neural foramen with foraminal widening and extension into the spinal canal, displacing the nerve roots to the left, with infiltration of the right paravertebral soft tissues and right psoas muscle. (C) Axial T2-weighted image at the L3 level showing near-complete occupation of the spinal canal with contralateral (left) foraminal extension. (D) Sagittal T1-weighted post-contrast image illustrating lytic destruction of the L3 vertebral body with collapse of the superior endplate and enhancing epidural tumor component.

Based on the clinical and radiological findings, the initial differential diagnoses included metastatic disease, plasmacytoma, lymphoma, and primary vertebral malignancy.

The patient underwent a decompressive laminectomy with subtotal tumor resection. Gross total resection was not feasible because of the extensive epidural and paravertebral extension of the lesion with involvement of adjacent vertebral structures. The surgical objective was therefore limited to adequate spinal cord decompression while minimizing the risk of neurological morbidity.

Histopathological examination of the resected specimen revealed diffuse infiltration of fibrous and osseous tissue by a monotonous lymphoid proliferation composed predominantly of small- to medium-sized cells. The tumor cells exhibited round to slightly irregular nuclei with finely dispersed chromatin, inconspicuous to small nucleoli, and scant to moderate pale cytoplasm. Mitotic figures were infrequent. Focal bone trabecular infiltration was observed, along with areas of ischemic necrosis (Figure 2).

**Figure 2:**
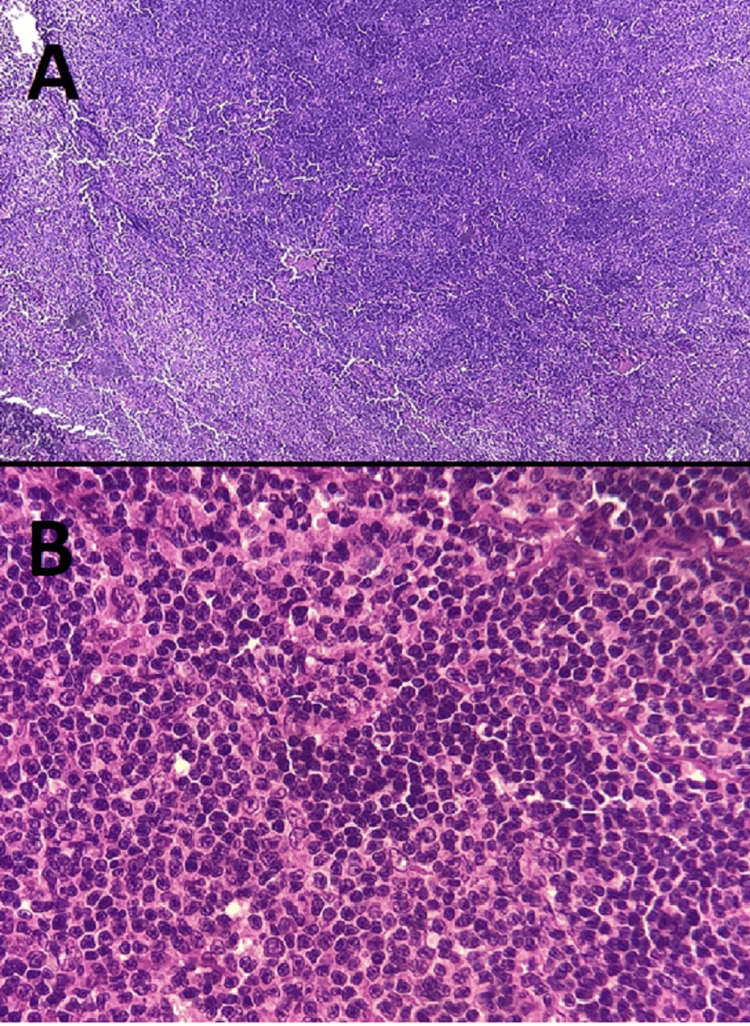
(A) Low-power magnification showing a diffuse lymphomatous infiltration with areas of nodular reinforcement (H&E, x4). (B) High-power magnification highlighting a proliferation of centrocyte-like cells: medium-sized neoplastic cells with vesicular nuclei, finely dispersed chromatin, and occasional reniform (kidney-shaped) nuclear contours (H&E, x40).

No well-formed germinal centers were identified. Occasional plasmacytic differentiation was noted.

Immunohistochemical analysis demonstrated diffuse expression of CD20, confirming B-cell lineage. The neoplastic cells were negative for CD5, CD10, and BCL6, arguing against mantle cell lymphoma and follicular lymphoma. BCL2 was diffusely expressed. CD23 highlighted scattered follicular dendritic cell meshworks but was negative in tumor cells. Importantly, CD138 expression was absent in the neoplastic lymphoid population, with staining restricted to rare reactive plasma cells, making plasmacytoma and lymphoplasmacytic lymphoma unlikely. In situ hybridization demonstrated kappa light-chain restriction with lambda negativity, confirming B-cell clonality. The Ki-67 proliferation index was approximately 15%, supporting an indolent lymphoid neoplasm. Taken together, the morphologic appearance and immunophenotypic profile were most consistent with extranodal marginal zone B-cell lymphoma (Figure 3).

**Figure 3:**
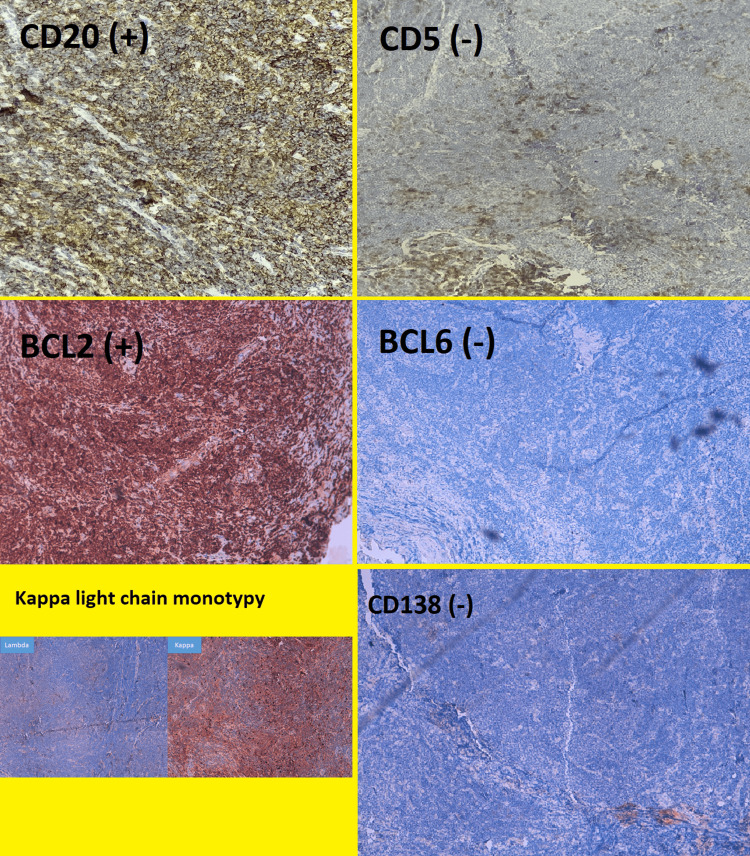
Immunophenotypic characterization of the marginal zone lymphoma. The tumor cells are CD20+ and BCL2+, while remaining negative for CD5, BCL6, and CD138. Note the clear Kappa light chain restriction (monotypicity), which is diagnostic of a clonal B-cell process.

Staging workup with brain and thoraco-abdomino-pelvic CT demonstrated postoperative regression of the lumbar mass without cerebral involvement. Additional findings included mild pleural effusion, imaging features of chronic liver disease with portal hypertension, and antropyloric thickening. Bone marrow biopsy showed no lymphoma infiltration.

Upper gastrointestinal endoscopy revealed grade II esophageal varices, erythematous gastritis, and ulcerative bulbitis. Gastric biopsies demonstrated mild chronic follicular gastritis with *Helicobacter pylori* infection, without atrophy, intestinal metaplasia, or dysplasia. Serology showed isolated hepatitis B core antibody (anti-HBc) positivity, with negative hepatitis B surface antigen (HBsAg) and hepatitis B surface antibody (anti-HBs), and negative HIV testing.

The patient was referred to radiation oncology. At presentation to our department after surgery, the patient was neurologically stable with preserved lower-limb strength and no sphincter dysfunction.

Given residual disease, adjuvant radiotherapy was delivered. Simulation CT (3-mm slices) was performed. The clinical target volume encompassed the initial MRI-defined tumor bed and residual disease with a 5-mm margin, including vertebral levels L2-L4. A 10-mm expansion generated the planning target volume. A total dose of 24 Gy in 12 fractions was prescribed. Treatment was planned using volumetric modulated arc therapy (VMAT) to optimize organ-at-risk sparing, particularly bowel dose constraints of V15 < 800 cc (Figure 4).

**Figure 4:**
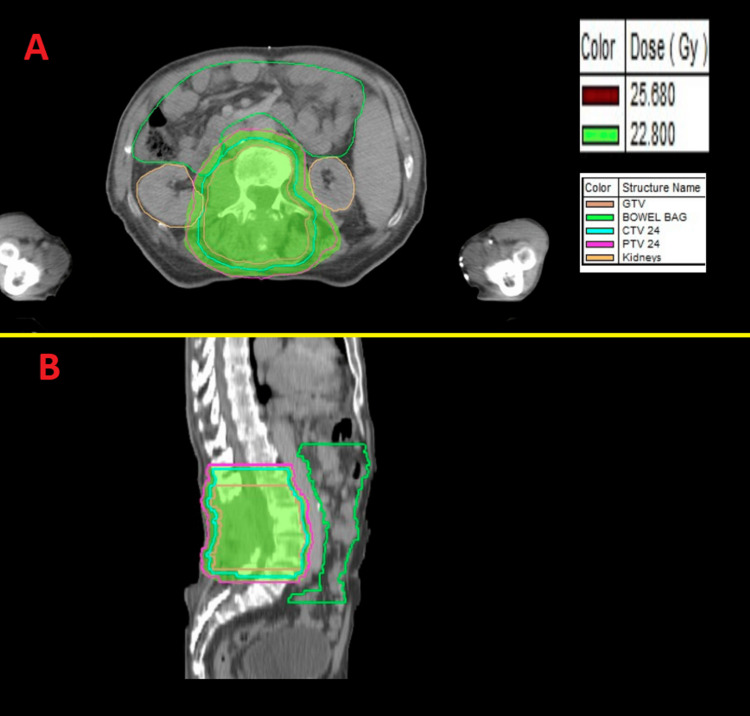
Volumetric modulated arc therapy (VMAT) radiotherapy treatment planning showing isodose distribution and bowel sparing. (A) Axial planning computed tomography (CT) demonstrating target volume coverage (gross tumor volume (GTV)/clinical target volume (CTV)/planning target volume (PTV)) with surrounding isodose lines and sparing of organs at risk, particularly the bowel bag and kidneys. (B) Sagittal planning CT illustrating conformal dose distribution encompassing vertebral levels L2–L4 with adequate target coverage and optimization of bowel dose constraints.

At the three-month follow-up, MRI demonstrated marked regression of the lesion with no evidence of recurrent spinal canal compression. Clinically, the patient remained neurologically intact with preserved ambulation and no treatment-related toxicity (Figure 5).

**Figure 5:**
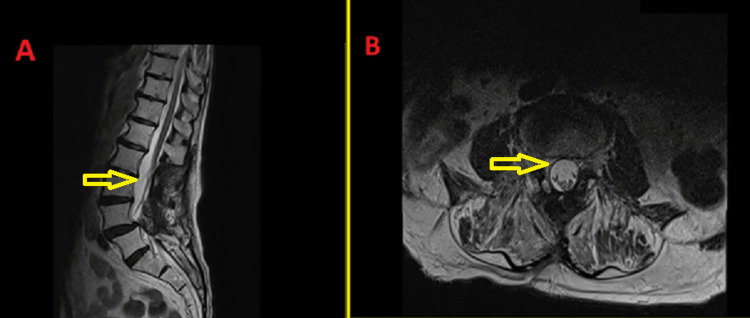
Follow-up lumbar spine MRI three months after radiotherapy. (A) Sagittal T2-weighted image demonstrating marked regression of the previously described hyperintense epidural and paravertebral lesion with restoration of the spinal canal. (B) Axial T2-weighted image confirming resolution of spinal canal compression and satisfactory canal dimensions.

## Discussion

Marginal zone lymphomas (MZLs) are indolent B-cell neoplasms derived from mature post-germinal center memory B cells located in the marginal zone of secondary lymphoid follicles. These cells play a key role in immune regulation and chronic inflammatory responses, a biological context that supports lymphomagenesis in the setting of persistent antigenic stimulation. As highlighted in the British Society of Haematology (BSH) guideline, MZL frequently arises in association with chronic infections or autoimmune disorders and demonstrates considerable biological and clinical heterogeneity [1].

Epidemiologically, MZL represents the third most common subtype of non-Hodgkin lymphoma in Western countries, accounting for approximately 15% of cases [3]. Extranodal MZL of mucosa-associated lymphoid tissue (MALT lymphoma) constitutes more than 60% of cases, followed by splenic MZL (approximately 20%) and nodal MZL (<10%). Incidence increases with age, supporting the hypothesis of cumulative exposure to etiological triggers.

According to the fifth edition of the WHO classification of hematolymphoid tumors, MZL comprises three principal subtypes: extranodal (MALT), splenic, and nodal MZL. Pediatric MZL and primary cutaneous MZL are now recognized as distinct entities [4]. This refined classification emphasizes the importance of tissue microenvironment and clinicopathologic correlation in diagnosis.

The pathogenesis of MZL is strongly linked to chronic antigenic stimulation. *Helicobacter pylori* is the best-established etiological agent in gastric MALT lymphoma, while other infectious agents, such as *Chlamydia psittaci*, *Campylobacter jejuni*, and *Borrelia burgdorferi*, have been associated with extranodal MZL in specific anatomical sites [5]. Hepatitis C virus infection has also been implicated, particularly in splenic and nodal forms [6]. Although our patient showed isolated anti-HBc positivity, no evidence of active viral hepatitis or systemic lymphomatous involvement was identified. In our patient, *Helicobacter pylori *infection was detected; however, a direct pathogenic link with spinal involvement cannot be established.

Histologically, EMZL is characterized by a heterogeneous population of small B cells, including centrocyte-like cells, monocytoid B cells, and variable plasmacytic differentiation. The growth pattern may be diffuse or vaguely nodular. Lymphoepithelial lesions are characteristic in epithelial organs but are not obligatory [7]. In our case, the lesion showed diffuse infiltration by small- to medium-sized lymphoid cells with low mitotic activity and focal bone involvement, consistent with an indolent B-cell lymphoma.

Immunophenotyping is essential for diagnosis, as no single marker is specific for MZL. According to BSH recommendations, immunohistochemistry primarily serves to exclude other small B-cell lymphomas [1]. MZL cells typically express pan-B markers (CD20, CD79a, and PAX5) and are negative for CD10, cyclin D1, and usually CD5. In the present case, the tumor cells were CD20 positive and negative for CD5, CD10, and BCL6, effectively excluding chronic lymphocytic leukemia/small lymphocytic lymphoma, mantle cell lymphoma, and follicular lymphoma. CD23 was negative in tumor cells. Importantly, CD138 was negative in neoplastic lymphoid cells, arguing against plasmacytoma or significant plasmacytic differentiation. Demonstration of kappa light-chain restriction confirmed clonality, and the low Ki-67 proliferation index (approximately 15%) supported an indolent phenotype. These integrated findings are fully consistent with EMZL.

Spinal and central nervous system involvement by MZL is rare. Most reported cases involve the dura, and management strategies are heterogeneous due to the low incidence of this entity. In a multicenter retrospective study of CNS- and dural-involved EMZL, favorable outcomes were reported following surgical resection combined with radiotherapy [8]. Our case similarly required an urgent decompressive laminectomy due to spinal cord compression, followed by adjuvant radiotherapy.

The role of radiotherapy in localized MZL is well established. The randomized phase III FoRT trial demonstrated superior local control with 24 Gy in 12 fractions compared with 4 Gy in 2 fractions in indolent B-cell lymphomas, including MZL [9]. After long-term follow-up, 24 Gy significantly reduced local progression rates, confirming it as the standard dose for curative-intent treatment, while 4 Gy is mainly reserved for palliation. Accordingly, our patient received 24 Gy, achieving good clinical recovery.

Population-based data further support the efficacy of radiotherapy in localized EMZL. In a large Surveillance, Epidemiology, and End Results (SEER) analysis of 7,961 patients with stage I EMZL, radiation therapy was associated with excellent long-term survival, comparable to the general population [10]. Surgery alone yielded inferior outcomes, whereas combined surgery and radiotherapy provided optimal results in selected cases. Only a limited number of spinal or epidural EMZL cases have been reported in the literature [11,12]. Most patients presented with progressive neurological symptoms related to epidural compression and were treated with combined surgical decompression and radiotherapy, achieving good neurological recovery and local disease control [11-14]. In our patient, surgery was necessary for decompression and diagnosis, and adjuvant radiotherapy was administered to optimize local control.

Overall, MZL is associated with a favorable prognosis. A prognostic index incorporating age ≥70 years, advanced stage, and elevated LDH identifies higher-risk patients [15]. Transformation to high-grade lymphoma occurs in fewer than 10% of cases and is associated with adverse features such as elevated lactate dehydrogenase (LDH) and incomplete response to initial therapy [16]. Modern treatment series confirm high response rates and durable survival in most patients [17]. In our case, despite advanced age, the absence of systemic disease and the indolent histologic profile suggest a favorable outlook.

Although the short-term neurological and radiological response observed in our patient is encouraging, the follow-up duration remains limited. Given the indolent nature of MZL and its potential for late relapse, durable local control and long-term progression-free survival cannot yet be assessed.

In summary, this case illustrates a rare presentation of EMZL as an extensive spinal and paravertebral mass causing medullary compression. Combined surgical decompression and standard-dose radiotherapy provided effective local disease control, consistent with current evidence-based recommendations. However, longer follow-up is required to evaluate the durability of response and long-term disease control in this indolent lymphoma subtype.

## Conclusions

Primary spinal EMZL is an exceptionally rare cause of spinal cord compression. Accurate diagnosis requires careful integration of imaging, histopathology, and immunophenotypic findings to exclude other indolent B-cell neoplasms and plasma cell disorders. In symptomatic spinal presentations, combined surgical decompression and radiotherapy may provide effective short-term neurological and local disease control.
